# IGF1 gene therapy in middle-aged female rats delays reproductive senescence through its effects on hypothalamic GnRH and kisspeptin neurons

**DOI:** 10.18632/aging.204360

**Published:** 2022-11-01

**Authors:** Franco Juan Cruz Dolcetti, Eugenia Falomir-Lockhart, Francisco Acuña, Macarena Lorena Herrera, Sofia Cervellini, Claudio Gustavo Barbeito, Daniela Grassi, Maria-Angeles Arevalo, María José Bellini

**Affiliations:** 1 Laboratorio de Bioquímica del Envejecimiento, Instituto de Investigaciones Bioquímicas de La Plata (INIBIOLP), Facultad de Ciencias Médicas, UNLP-CONICET, La Plata, Argentina; 2 Laboratorio de Histología y Embriología Descriptiva, Experimental y Comparada, Facultad de Ciencias Veterinarias, Universidad Nacional de La Plata, La Plata, Argentina; 3 Instituto de Farmacología Experimental de Córdoba-CONICET, Departamento de Farmacología, Facultad de Ciencias Químicas, UNC-CONICET, Córdoba, Argentina; 4 Department of Anatomy, Histology and Neuroscience, Autonomous University of Madrid, Madrid, España; 5 Instituto Cajal, CSIC, Madrid, España; 6 Centro de Investigación Biomédica en Red de Fragilidad y Envejecimiento Saludable (CIBERFES), Instituto de Salud Carlos III, Madrid, España

**Keywords:** IGF1, gene therapy, reproductive senescence, GnRH, kisspeptin, microglia

## Abstract

The process of aging is the result of progressive loss of homeostasis and functional body impairment, including the central nervous system, where the hypothalamus plays a key role in regulating aging mechanisms. The consequences of aging include a chronic proinflammatory environment in the hypothalamus that leads to decreased secretion of gonadotropin-releasing hormone (GnRH) and impairs kisspeptin neuron functionality.

In this work, we investigated the effect of insulin-like growth factor 1 (IGF1) gene therapy on hypothalamic kisspeptin/GnRH neurons and on microglial cells, that mediate the inflammatory process related with the aging process. The results show that IGF1 rats have higher kisspeptin expression in the anteroventral periventricular (AVPV) nucleus and higher immunoreactivity of GnRH in the arcuate nucleus and median eminence. In addition, IGF1-treated animals exhibit increased numbers of Iba1^+^ microglial cells and MHCII^+^/Iba1^+^ in the AVPV and arcuate nuclei. In conclusion, IGF1 gene therapy maintains kisspeptin production in the AVPV nucleus, induces GnRH release in the median eminence, and alters the number and reactivity of microglial cells in middle-aged female rats. We suggest that IGF1 gene therapy may have a protective effect against reproductive decline.

## INTRODUCTION

Aging is the result of the progressive loss of homeostasis and functional body impairment affecting the whole body, including the central nervous system (CNS). The hypothalamus has been described to be the key brain area responsible for the regulation of whole-body aging processes, by modulating neuroendocrinological as well as inflammatory events [1–3]. The inflammatory environment characteristic of the aged brain is caused by activation of glial cells, mainly microglia [4]. These cells undergo genotypical and morphological changes, increase the secretion of inflammatory mediators such as cytokines and free radicals, as well as neurotrophins, growth factors and extracellular matrix proteins [5].

Several studies report that neuroinflammation leads to reduced gonadotropin-releasing hormone (GnRH) secretion, which is associated with multiple aging-related physiological changes, including bone loss, skin atrophy, muscle weakness, and memory loss. Indeed, GnRH administration amend aging-impaired neurogenesis and decelerates aging in mice [3, 6]. In addition, the same authors also describe that inhibition of NF-κB-directed immunity, specifically in hypothalamic microglia cells, has an anti-aging effect [3].

GnRH is a bioactive decapeptide secreted by nerve terminals in the median eminence, that travels through the hypophyseal-portal blood vessels to bind the GnRH receptor (GnRH-R) in the gonadotropic cells of the pituitary. Stimulation of gonadotropic cells by GnRH is required for the biosynthesis of luteinizing hormone (LH) and follicle-stimulating hormone (FSH), which control fertility and reproduction. GnRH secretion is regulated by hormonal and environmental signals such as kisspeptin. This peptide plays a critical role in controlling the onset of puberty and reproductive function in adulthood [7–11]. There are two populations of kisspeptin neurons, one in the anteroventral periventricular nucleus (AVPV) and one in the arcuate nucleus (Arc), that are targets of positive and negative feedback regulation of estrogen, respectively. Kisspeptin neurons in the AVPV are thought to mediate the positive feedback effect of estrogen that causes the GnRH/LH surge during the periovulatory period, whereas kisspeptin neurons in the Arc are thought to regulate the negative feedback effect of estrogen on tonic GnRH/ LH secretion [12–15].

Aging female rats transition from regular to irregular estrus cycles, constant estrus, and finally to an anestrus stage. Changes within the hypothalamic-pituitary-ovarian axis, manifested by altered secretion of neurotransmitters, altered secretion of pituitary hormones and altered follicular development and steroid content, lead to the final cessation of reproductive cycles. Middle-aged rats with constant estrus (CE) (12 months old) present elevated estrogen levels compared to young, cyclic rats [16].

These processes lead to reproductive senescence associated with an increase in circulating cytokines and proinflammatory markers produced by microglial cells. Indeed, several studies describe that hypothalamic and systemic inflammation affect kisspeptin neurons, which are responsible for regulating GnRH neurons [17–19]. However, the precise role of kisspeptin neurons during female reproductive senescence remains unclear. Some evidence suggests that among the groups of neurons involved in reproductive control, kisspeptin neurons in the AVPV hypothalamic nucleus are probably among the earliest to undergo aging processes and thus are involved in triggering early reproductive decline [20]. The mechanism by which age-related neuroinflammation regulates kisspeptin and GnRH secretion is still poorly understood.

IGF1 is a neurotrophic factor with an outstanding neuroprotective action in the central nervous system. Previous studies of our group showed that intraparenchymal hypothalamic IGF1 gene therapy was capable to prolong cyclicity in middle-aged Sprague Dawley female rats [21]. Indeed, we have demonstrated that intracerebroventricular IGF1 gene therapy restores motor performance [22] and generates cognitive and morphological changes in the dorsal hippocampus in senile rats [23]. In addition, we have reported that IGF1 gene therapy modifies microglia number and phenotype senile rats [24] and decreases astrocytic inflammatory response *in vitro* [25], supporting the extensive idea that IGF1 plays a potent anti-inflammatory effect [26–28].

The aim of the present study is to investigate the effect of IGF1 gene therapy on estrous cycle, kisspeptin and GnRH neurons, and microglial cells in middle-aged female rats. Our data indicate that IGF1 gene therapy prolongs cyclicity in middle-aged rats by modulating kisspeptin/GnRH secretion in the hypothalamus and altering microglial cell number and reactivity. Based on our findings, we propose IGF1 gene therapy to delay reproductive senescence as a potential strategy to optimize lifespan and combat age-related health problems in women.

## RESULTS

### IGF1 gene therapy modifies the frequency and maintains the cyclicity in 12-month-old female rats

We primarily calculated estrus cycle frequency as the number of cycles per week for the pre-treatment period (day −30 to 0) and the post-treatment period (day 0 to 120) based on vaginal smears. A cycle is considered regular if it has a 24-hour proestrus (P), a 24-hour estrus (E), and a 48–72-hour metestrus (M) (also known as diestrus I) and diestrus (D) (also known as diestrus II). Cycles that not fulfill the above requirements are defined as irregular. Constant estrus (CE) was defined as up to 5 consecutive days in estrus (E).

**Figure 1:**
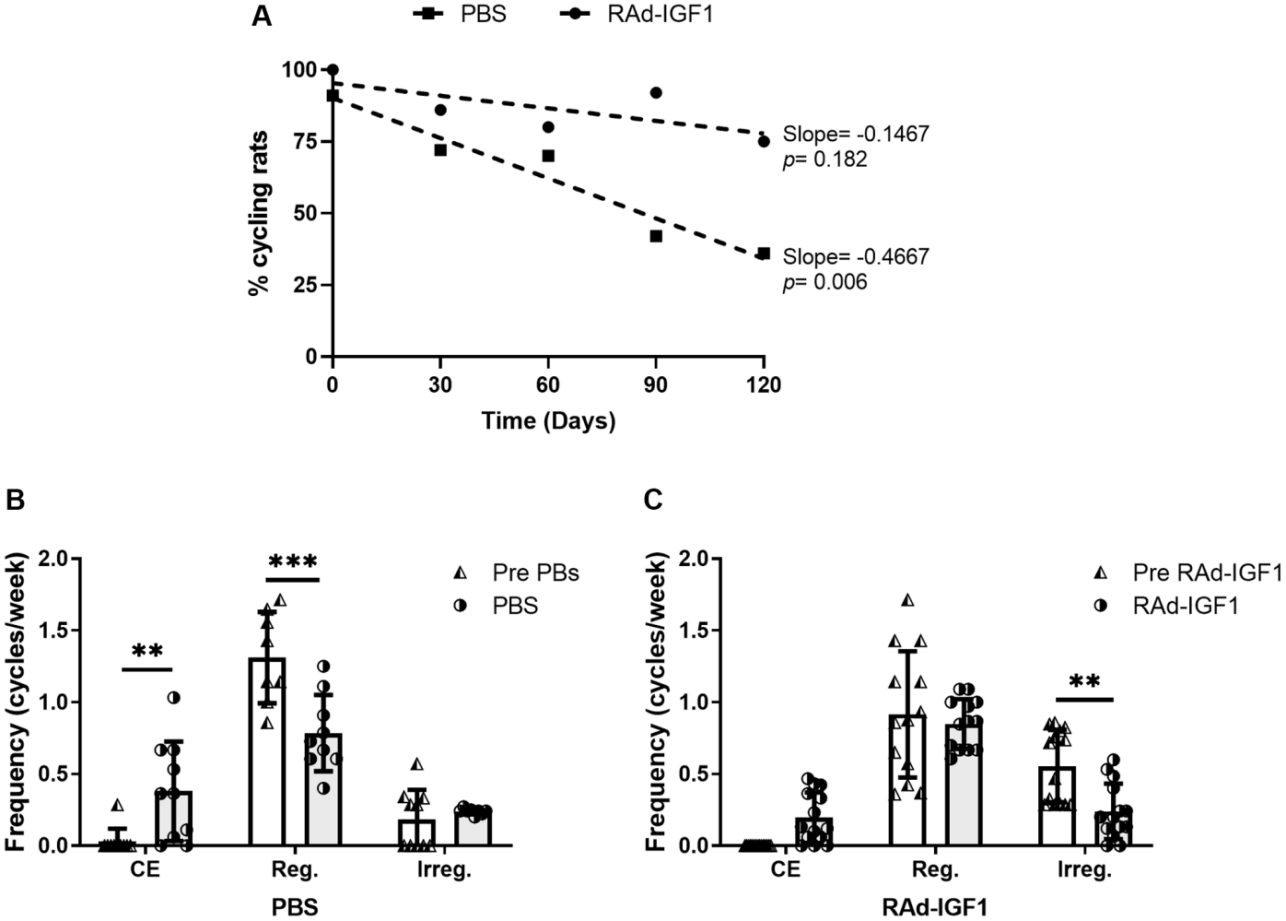
**IGF1 gene therapy effect on cyclicality status in MA female rats.** (**A**) IGF1 gene therapy effect on the proportion of cycling rats. Significant (*p* < 0.05) negative slope indicate a decrease of cycling rat in control group (PBS) along the experiment. (**B** and **C**) IGF1 gene therapy effect on the frequency of cycles. Rats had their vaginal cytology assessed daily from day −30 to 120. The frequencies were calculated as the number of cycles per week for the pretreatment period (day −30 to 0) and the treatment period (day 0 to 120). Abbreviations: CE: Constant Estrus (5 days of Estrus); Reg: Regular (1 day of Proestrus, 1 day of Estrus, 2–3 days of Metestrus/Diestrus); Irreg: Irregular. Error bars represent SD (N_RAd-IGF1_ = 13; N_PBS_ = 9). ANOVA followed by the Bonferroni's multiple comparisons test was used. Asterisks indicate significant (^**^p < 0.005; ^***^p < 0.001) differences vs. respective pretreatment value. Figure 1B post hoc power (1-β) analysis: 0.907 (Factor A); 0.953 (Factor B). Figure 1C post hoc power (1-β) analysis: 0. 985 (Factor A); 0. 994 (Factor B).

Control animals showed a negative correlation between cycling rats and time in PBS animals (slope = −0.1467, *F*_(1, 3)_ = 48.68, *p* = 0.006, R^2^ = 0.942). These results indicate a decrease in the proportion of cycling rats compared to animals before PBS injection. We did not detect any correlation in RAd-IGF1 treated animals (slope = −0.4667, *F*_(1, 3)_ = 3.00, *p* = 0.182, R^2^ = 0.500). Thus, no significant differences were detected in RAd-IGF1 animals compared to the previous RAd-IGF1 injection (Figure 1).

In addition, RAd-IGF1-treated animals did not show significant differences in either the frequency of CE cycles or the frequency of regular cycles between the beginning and end of the experiment. Moreover, this group showed a significant decrease in the frequency of irregular cycles. In the control group, a significant increase in the frequency of constant estrus cycles and, consequently, a decrease in the frequency of regular cycles was observed (Figure 1). These results suggest that IGF1 gene therapy maintains cyclicity in rats.

### IGF1 modulates ovarian follicles and corpora lutea

Histological descriptive analysis of ovaries was performed in cycling and not cycling rats in both RAd-IGF1 and PBS groups.

**Figure 2:**
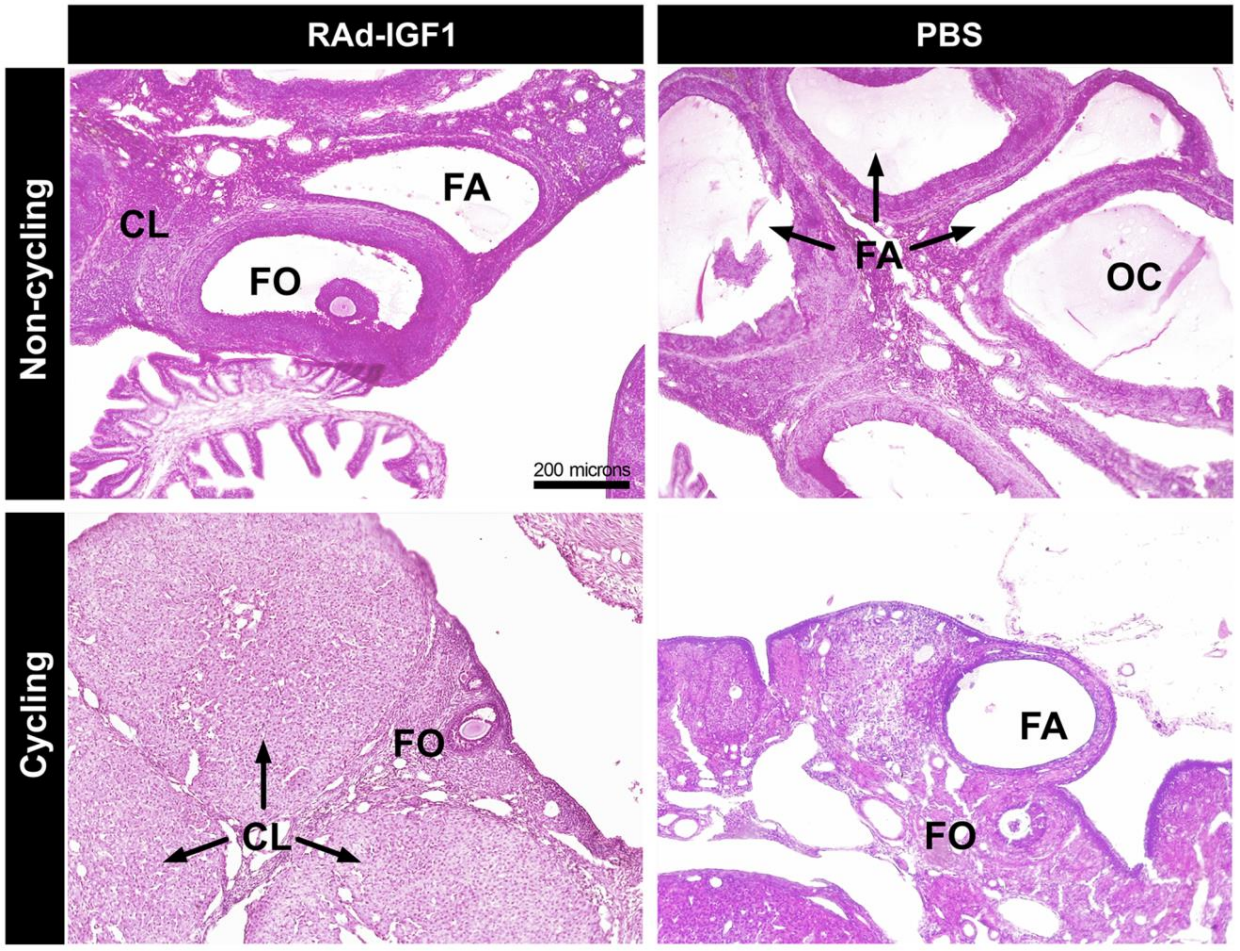
**Histology of representative ovaries from control (PBS) and experimental (RAd-IGF1) 12-months-old MA Sprague Dawley rats.** Ovarian sections from non-cycling (above) and cycling rats (below). Abbreviations: CL: corpora lutea; FA: atretic follicles; FO: ovarian follicles; OC: ovarian cysts. Total magnification of 100× (scale bar: 200 microns).

In the cycling rats, we observed follicles at different stages of development, corpora lutea (CL) and atretic follicles in both groups. However, in the ovaries of RAd-IGF1-treated rats, the number of atretic follicles was lower and the number of follicles at different developmental stages was higher (Figure 2).

The ovaries of the non-cycling control females showed hardly any CL, fewer growing follicles and fewer ovarian cysts. No ovarian cysts were observed in the RAd-IGF1 group, and the ovaries had similar characteristics to those of the RAd-IGF1 cycling rats, but with a lower number of ovarian follicles and corpora lutea.

### IGF1 gene therapy does not modify E2 and LH levels in rat serum

**Figure 3:**
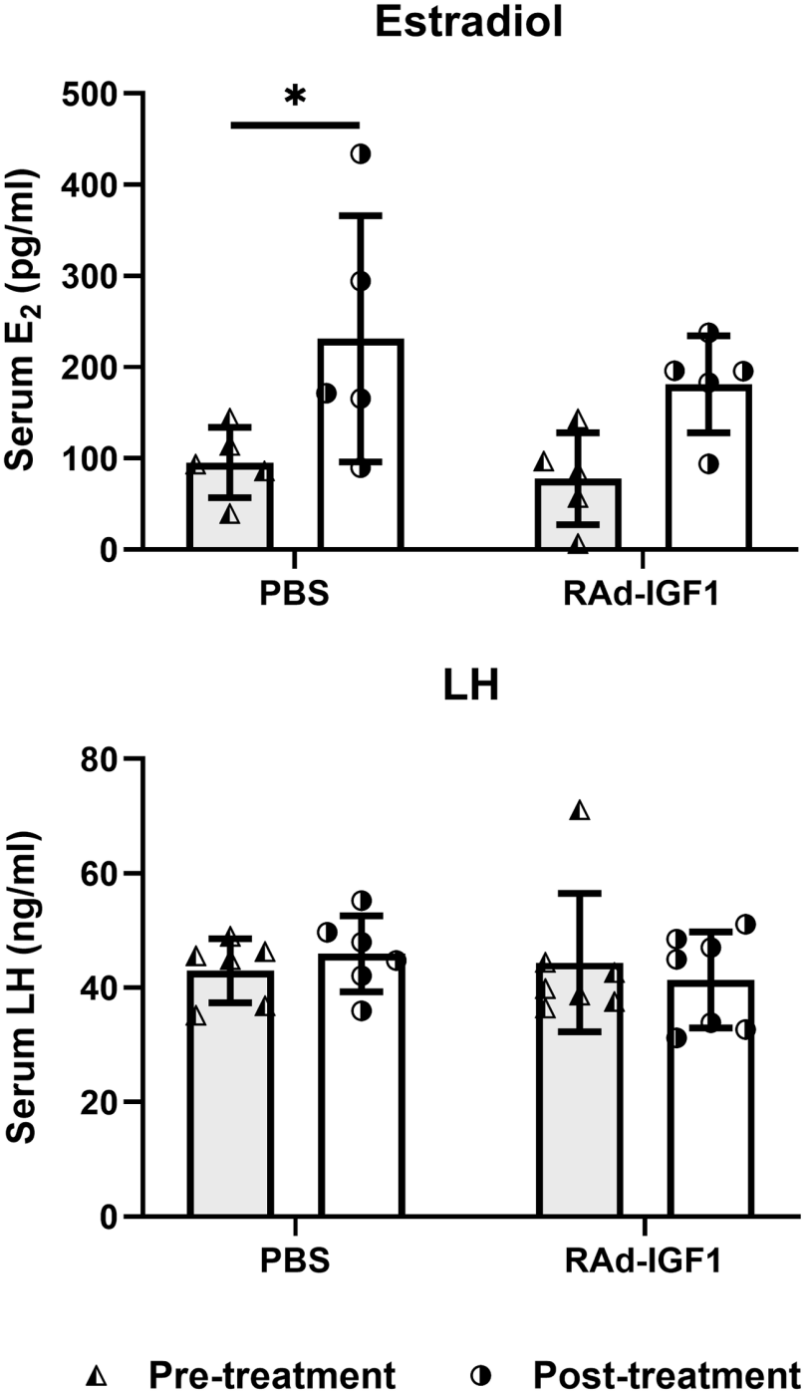
**Serum hormone levels in M-A rats before and after IGF1 gene therapy.** Blood samples were collected from the tail veins at the beginning (pre-treatment) and at the end of the study. Column height and bar above represents mean and SD respectively (Estradiol: N_RAd-IGF1_ = 5; N_PBS_= 5. LH: N_RAd-IGF1_ = 7; N_PBS_ = 6). ANOVA followed by the Bonferroni’s multiple comparisons test was used. Asterisks indicate significant (^*^p < 0.05) differences vs. pretreatment value. Figure 3_Estradiol_ post hoc power (1-β) analysis: 0. 5592 (both factors); Figure 3_LH_ post hoc power (1-β) analysis: 0. 6834 (both factors).

Serum levels of E2 and LH were measured 3 days before the experimental treatments (day-3) and at the end of the experiment (day 120). As can be seen in Figure 3, only the control group treated with PBS showed a significant increase in E2 levels at the end of the treatment (*F*_(1, 16)_ = 11.42, *p* = 0.0038), while no significant differences were observed in LH levels (*F*_(1, 24)_ = 0.19, *p* = 0.6667). The data suggest that IGF1 gene therapy prevents decline or at least restores hormone levels to pre-treatment levels.

### IGF1 gene therapy modulates kisspeptin and GnRH immunoreactivity in the AVPV nucleus and in the ME

Given the data from ovarian histological analysis, we decided to investigate the effect of RAd-IGF1 on GnRH and kisspeptin neurons, involved in the regulation of the hypothalamic-pituitary-gonadal axis.

**Figure 4:**
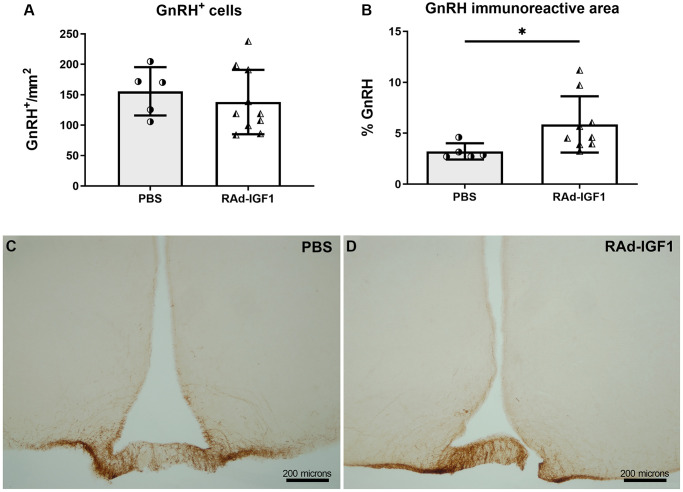
**IGF1 gene therapy effect on the GnRH immunopositive cells.** (**A**) Quantification of GnRH immunopositive cells (N_RAd-IGF1_ = 10; N_PBS_ = 5). (**B**). GnRH immunopositive fiber density measured by immunoreactive area (N_RAd-IGF1_ = 9; N_PBS_ = 5). Error bars represent SD. Two-tailed *t*-test (**A**) or Mann Whitney test (**B**) was used. Asterisks indicate significant (^**^p < 0.01) differences. (**C** and **D**) Immunohistochemistry for GnRH of control (PBS) and experimental (RAd-IGF1) rat’s brain slides. Total magnification of 100× (scale bars: 200 microns).

GnRH-immunoreactive neuronal somas were quantified in the preoptic area (PoA) and their axonal projections in the arcuate nucleus (Arc) and median eminence (ME). As shown in Figure 4A, no significant differences in the number of neuronal bodies were detected between groups (*t*_(13)_ = 0.6543, *p* = 0.5243). However, a significant increase in the immunoreactive area in the Arc and ME was observed in RAd-IGF1 group (Mann–Whitney *U* = 4*, n_1_* = 5 *n_2_* = 8*, p* = 0.0186) (Figure 4B). Representative examples of GnRH immunoreactivity are shown in Figure 4C and 4D.

**Figure 5:**
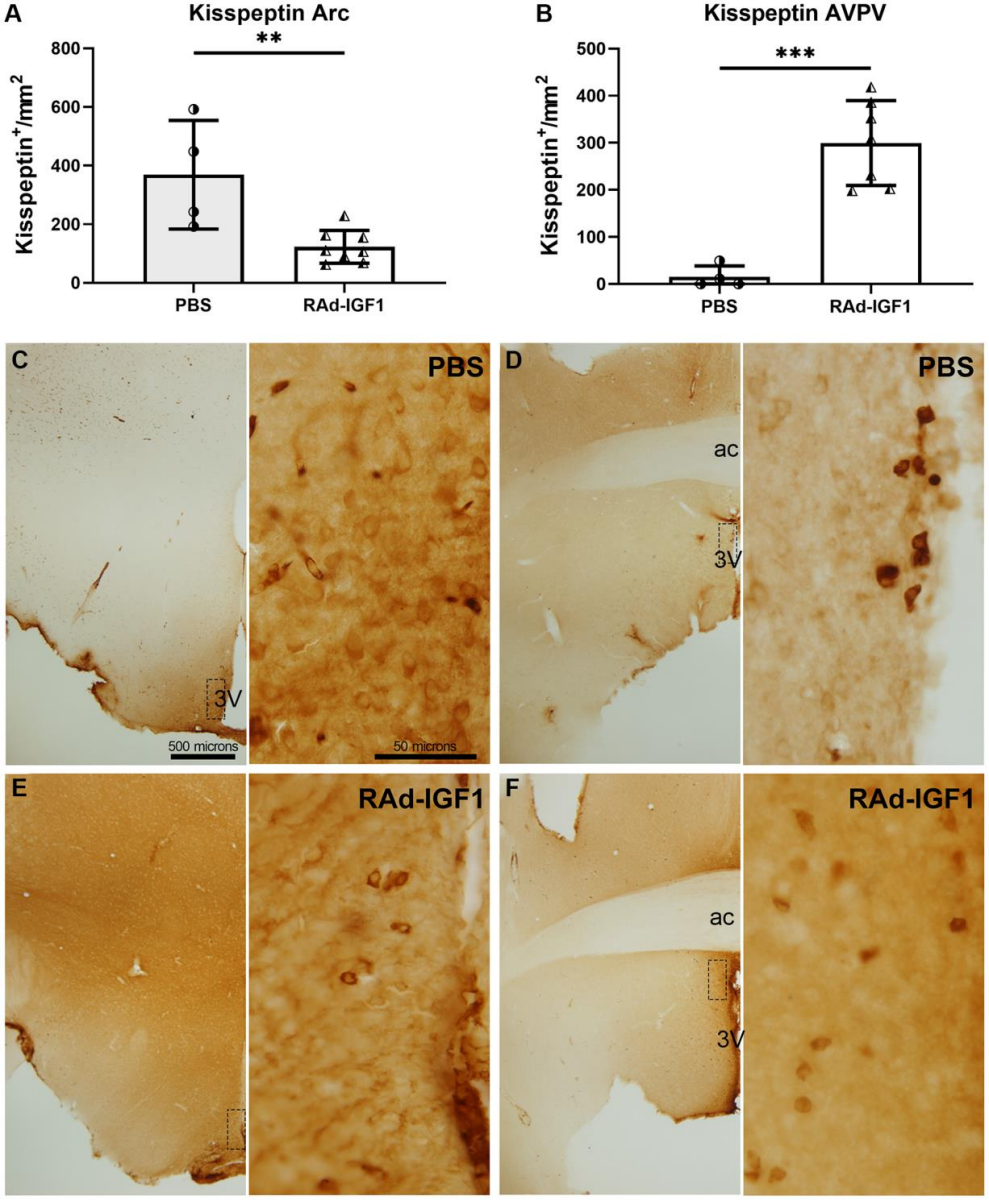
**IGF1 gene therapy effect on the kisspeptin immunopositive cells.** (**A** and **B**) Quantification of kisspeptin immunopositive cells. Error bars represent SD (N_RAd-IGF1_ = 7; N_PBS_ = 4). Two-tailed *t*-test was used. Asterisks indicate significant (^**^p < 0.005; ^***^p < 0.001) differences. Figure 5A post hoc power (1-β) analysis: 0.7533; Figure 5B post hoc power (1-β) analysis: 0.9999. (**C**–**F**) Immunohistochemistry for kisspeptin of control (PBS) and experimental (RAd-IGF1) rat’s brain slides at a magnification of 40× (scale bar: 500 microns), with insets at a magnification of 600× (scale bar: 50 microns). 3V: third ventricle; ac: anterior commissure.

Immunopositive kisspeptin cells were quantified in the anteroventral periventricular nucleus (AVPV) and arcuate nucleus (Arc). RAd-IGF1 animals showed a significant decrease in the number of immunopositive kisspeptin cells in the arcuate nucleus (*t*_(10)_ = 3.595, *p* = 0.0049) (Figure 5A) and an increase in the AVPV nucleus (*t*_(9)_ = 6.059, *p* = 0.0002) (Figure 5B) compared to the control group. Representative examples of kisspeptin immunoreactivity are shown in Figure 5C–5F.

Overall, our results suggest that IGF1 gene therapy modulates the hypothalamic kisspeptin system and GnRH expression in the median eminence.

### IGF1 gene therapy modifies the number and reactivity of microglia cells in the hypothalamus

**Figure 6:**
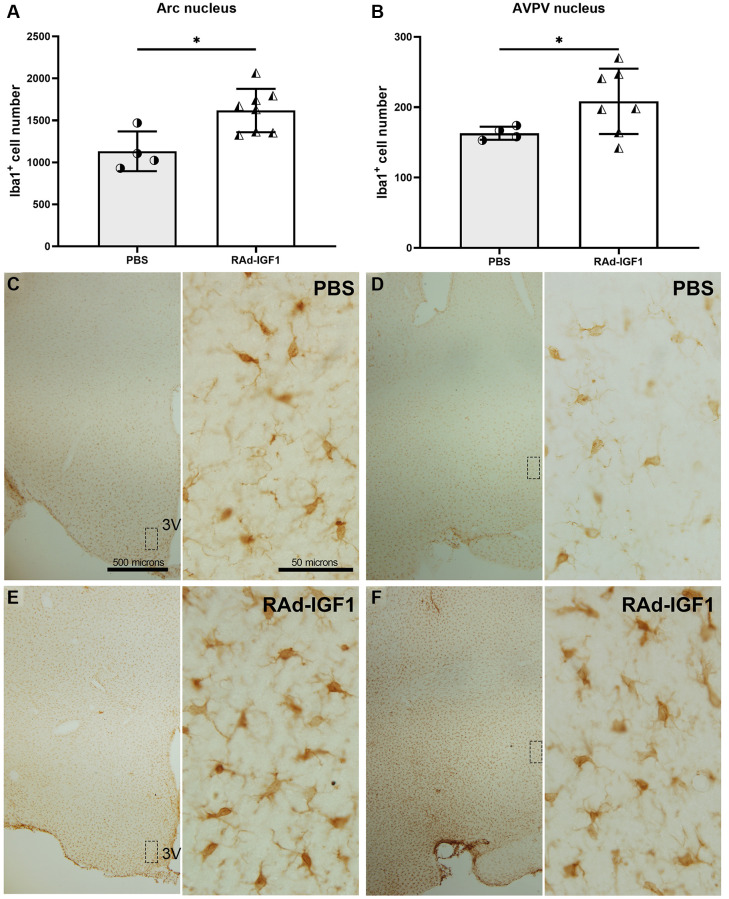
**IGF1 gene therapy effect on the Iba1 immunopositive cells.** (**A** and **B**) Quantification of Iba1 immunopositive cells. Error bars represent SD (Arcuate: N_RAd-IGF1_ = 8; N_PBS_ = 4. AVPV: N_RAd-IGF1_ = 7; N_PBS_ = 4). *t* test with Welch’s correction was used. Asterisks indicate significant (^*^p < 0.05) differences. Figure 6A post hoc power (1-β) analysis: 0.8211; Figure 6B post hoc power (1-β) analysis: 0.4876. (**C**–**F**) Immunohistochemistry for Iba1 of control (PBS) and experimental (RAd-IGF1) rat’s brain slides at a magnification of 40× (scale bars: 500 microns), with insets at a magnification of 600× (scale bars: 50 microns). Abbreviation: 3V: third ventricle.

To characterize hypothalamic immunity/inflammation in middle-aged rats, we profiled microglia in the hypothalamus using immunostaining. The data showed an increase in the number of immunoreactive Iba1 cells in the AVPV and Arcuate nuclei in RAd-IGF1 treated animals compared to the PBS group (Arcuate nucleus: *t*_(6.65)_ = 3.249, *p* = 0.0151; AVPV nucleus: *t*_(6.81)_ = 2.499, *p* = 0.0420) Figure 6A, 6B. Representative images of Iba1 immunoreactivity are shown in Figure 6C–6F.

**Figure 7:**
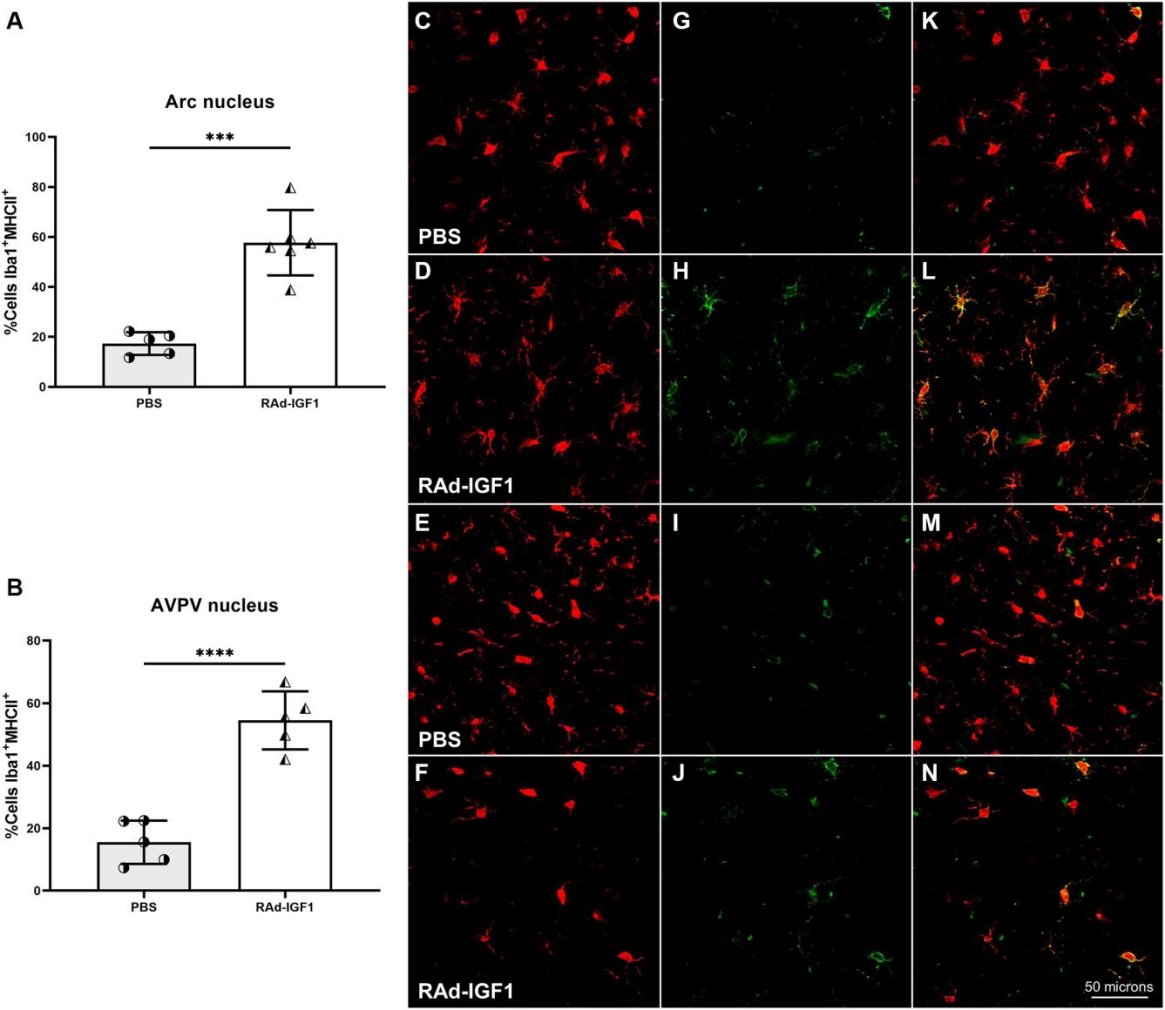
**IGF1 gene therapy effect on the activation state of microglia.** (**A** and **B**) Percentage of microglia presenting double immunostaining for Iba1 and MHCII. Error bars represent SD (Arcuate: N_RAd-IGF1_ = 6; N_PBS_ = 5. AVPV: N_RAd-IGF1_ = 5; N_PBS_ = 5). Two-tailed *t*-test was used. Asterisks indicate significant (^***^p < 0.0005; ^****^p < 0.0001) differences. Iba1 cells (**C**–**F**), MHCII cells (**G**–**J**) and colocalization of both markers (**K**–**N**) (Total magnification of 200×; scale bars: 50 microns). Figure 7A post hoc power (1-β) analysis: 0.99997; Figure 7B post hoc power (1-β) analysis: 0.999997.

Microglial phenotypes are divided into M1 [29], which elicits neuroinflammatory responses, and M2 [30], which is thought to promote tissue repair function. This latter phenotype is subdivided into M2a, M2b, and M2c [31]. To determine the activation state of microglia, we examined the proportion of microglia expressing MHCII, a marker that might be present in the M1 or M2b phenotype, in both AVPV and Arcuate nuclei. Interestingly, compared with the control group, RAd-IGF1-treated animals had a higher proportion of double immunopositive Iba1/MHCII cells in both nuclei compared to the control group (Figure 7A and 7B) (Arcuate nucleus: *t*_(9)_ = 6.520, *p* = 0.0001; AVPV nucleus: *t*_(8)_ = 7.522, *p* < 0.0001). Representative example of Iba1/MHCII double immunostaining is shown in Figure 7C–7N.

### IGF1 gene therapy modifies the number and senescent phenotype of hypothalamic astrocytes

To assess the effect of IGF1 gene therapy on astrocyte number and senescent phenotype, we performed double immunostaining with GFAP (as a marker for total astrocytes) and β-galactosidase as a marker for senescence. The data showed a higher number of GFAP immunopositive cells in the RAd-IGF1 group compared with the control group (Arcuate nucleus: *t*_(11.33)_ = 2.496, *p* = 0.03; AVPV nucleus: *t*_(8.64)_ = 2.409, *p* = 0.04). When double-positive cells were analyzed, the data showed a higher percentage in non-treated rats compared with the RAd-IGF1 group in the arcuate nucleus (*t*_(6.01)_ = 2.641, *p* = 0.04), suggesting that IGF1 gene therapy altered the aging phenotype of astrocytes in the hypothalamus (Supplementary Figure 1).

## DISCUSSION

In rats, the state of reproductive senescence is characterized by irregular estrous cycles. Rats go through a constant estrus cycle, with persistent vaginal cornification, until eventually becoming anestrus. Cycle frequency is used as an index of reproductive capacity, with a high frequency of regular cycles indicating rats with normal ovulatory activity and a high frequency of constant estrus phase indicating rats with ovulatory activity cessation. At 9 months of age, our Sprague Dawley rat colony began to have irregular cycles, followed by a prevalence of persistent estrus from 10–11 months of age [21]. Our goal was to use intrahypothalamic IGF1 gene therapy to delay reproductive senescence, understand how it affects kisspeptin/GnRH neurons, and modulate the microglia-mediated neuroinflammatory environment. Our results indicate that IGF1 gene therapy prolongs cyclicality in middle-aged female rats, confirming Rodriguez et al. findings [21]. Our findings support the hypothesis that this effect is triggered by a modification in kisspeptin/GnRH production, which leads to an increase in circulating systemic GnRH and a change in ovary structure and hormone levels.

Our results revealed several differences in the histological structure of ovaries between cycling and non-cycling rats. Indeed, the ovaries from cycling rats treated with IGF1 gene therapy showed more mature and less atretic follicles compared to control animals, whereas non-cycling control animals had large ovarian follicular cysts and scarce corpora lutea. This last observation is consisted with a sustained estradiol secretion described in anovulatory middle-aged rats [32]. The presence of follicles in different stages of development as well as better-preserved ovaries in RAd-IGF1 cycling rats strongly suggests that the IGF1 gene therapy delayed anovulation in our rat colony. In addition, when IGF1 animals are compared to control rats, hormonal data show a decrease in estradiol levels, which is consistent with previous research [16, 33, 34] Previous studies also have shown that IGF1 gene therapy can lead to an increase in the levels of LH [21] but we did not observe any change in our model. This could be due to a number of factors, including the observed differences in basal levels of LH. The data from Rodríguez et al. showed very low LH values compared with ours. In addition, the pretreatment IGF1 group had lower values than the intact pretreatment group, which could exacerbate the difference in the IGF1 group observed by these authors.

To investigate if IGF1 gene therapy can modify the hypothalamic GnRH system, we analyzed GnRH and kisspeptin immunoreactivity in the PoA, ME and AVPV, arcuate nucleus, respectively. The data showed a decrease in the number of immunopositive kisspeptin cells in the arcuate nucleus and an increase in the AVPV nucleus in RAd-IGF1 rats. These results are consistent with previous studies describing that positive sex-steroid feedback in rodents seems to be mediated via kisspeptin neurons in the AVPV region and negative sex-steroid feedback via the arcuate kisspeptin/Neurokinin B/Dynorphin neurons (KNDy) [12]. Thus, the decrease we observed in the arcuate nucleus could be related with still functional ovaries. Moreover, additional studies have showed that, in ovariectomized rodents, there is an increase in kisspeptin expression in the arcuate nucleus and a decrease in the rostral areas [13, 14].

Although our results suggest that arcuate kisspeptin neurons did not respond to the observed increased estrogen levels, this is consistent with previous reports describing that kisspeptin neurons in rodent rostral areas exhibit a decrease in estrogen receptor-α levels along with cellular senescence that increases with age. Moreover, these cells are thought to be among the first to undergo aging processes and are therefore involved in the onset of early reproductive decline [20]. Therefore, our results could be related to a higher susceptibility to aging and the absence of a kisspeptin neurons population response in the arcuate nucleus. Another novel finding is the higher kisspeptin expression in the AVPV nucleus in rats treated with RAd-IGF1, which would indicate the maintenance of the kisspeptin system in middle-aged rats.

We demonstrated that the number of immunopositive GnRH cells did not change with IGF1 gene therapy. This result is consistent with other reports describing that GnRH cell numbers do not change with age and that GnRH neurons, unlike kisspeptin neurons, do not undergo a process of cellular senescence [20, 35]. However, we observed a greater GnRH immunoreactivity in the arcuate nucleus and in the median eminence in RAd-IGF1 rats. Other studies have shown, both *in vitro* and *in vivo*, that continuous exposure to kisspeptin causes desensitization in the hypothalamus-pituitary-gonadal axis [36–39]. This could explain the decrease in GnRH immunoreactivity observed in the control group, although kisspeptin expression was increased in the arcuate nucleus.

With the purpose to identify possible changes in the inflammatory state of the hypothalamus, we decided to investigate if IGF1 gene therapy may modify the number of total microglia and activated macrophage/microglia cells in the arcuate and AVPV nuclei. For activated macrophage/microglia cells, we use MHCII as a marker. It is largely accepted that MHCII is a hallmark of activated cells; and enables antigen presentation in presence of costimulatory molecules [40].

Our data revealed that IGF1 gene therapy increased the number of total Iba1 immunopositive cells and double immunopositive Iba1/MHCII cells in both arcuate and AVPV nuclei. Although MHCII is expressed in both M1 and M2 microglia, we postulate that its increase is related with M2b microglial phenotype. This phenotype remains the least understood. It is characterized by the absence of M2-specific markers such as Arg1, YM1 or FIZZ1 and higher levels of MHCII and CD86. Some authors suggest that M2b cells also express anti-inflammatory markers and that M2b may be a potential regulator or initiator of the M2 response in general [31, 41].

Further studies need to be performed in the future to fully confirm our hypothesis. Overall, our data suggest that IGF1 gene therapy alters the inflammatory environment present in the hypothalamus of middle-aged rats by modifying the number and profile of microglial cells. These cells probably interact with kisspeptin neurons in the AVPV, leading to an increase in GnRH secretion, prolonging cyclicity in middle-aged rats and preserving ovarian morphology. This is supported by Zhang et al. [3] that revealed that microglial IKK-β and NF-κB inhibit GnRH to mediate ageing-related hypothalamic GnRH decline.

In conclusion, IGF1 gene therapy maintains kisspeptin production in AVPV and induces GnRH release in the median eminence and modifies microglia cells number and reactivity. Our findings prompt us to postulate that IGF1 gene therapy, implemented before the first signs of reproductive cessation, has a protective effect against the reproductive decline. It postpones the appearance of aging signs in the hypothalamus and delays reproductive senescence, producing a longer maintenance of the cyclicity and ovarian characteristics. In light of these observations, more work has to be done in order to elucidate the mechanism underlying IGF1 effect, who showed that microglial IKK-β and NF-κB inhibit GnRH to mediate the age-related decline in hypothalamic GnRH. In conclusion, IGF1 gene therapy maintains kisspeptin production in the AVPV and induces GnRH release in the median eminence and modifies microglial cells number and reactivity. Our findings prompt us to postulate that IGF1 gene therapy implemented before the first signs of reproductive cessation has a protective effect against reproductive decline. It postpones the appearance of aging signs in the hypothalamus and delays reproductive senescence, resulting in longer maintenance of cyclicity and ovarian characteristics. In light of these observations in future work we will investigate the mechanism underlying IGF1 action.

### Conclusions and future perspectives

This study was performed on a single time point, we need to further investigate if the effects of IGF1 gene therapy are maintained beyond 12 months. In future work, it will be interesting also to investigate if hormonal levels and ovarian response are affecting behavioral tasks. Moreover, in order to delve in the inflammatory status of the hypothalamus, it will be key to measure cytokines’ mRNA expression.

We postulate that prolonged cyclicity is related with an increase in GnRH secretion and with kisspeptin/microglia interaction on the basis of classic measurements. In future studies, it would be important to delve into the mechanisms that mediate these effects, like inhibition of GnRH secretion and microglia functions, and also delve in the characterization of inflammatory environment and microglia phenotype. It will be also interesting to measure the expression of classic and non-classic estrogen receptors as well as mRNA expression or total protein expression of kisspeptin, which could support our work.

## MATERIALS AND METHODS

### Animals

Seven-months old female Sprague-Dawley rats, belonged to our animal facilities, were used. Animals were housed in a temperature-controlled room (22 ± 2°C) on a 12-hour light, 12-hour dark cycle. Food and water were available *ad libitum*, with a standard chow diet containing 69.5% carbohydrates, 5.6% of fat, and 24.9% protein (Asociación de Cooperativas Argentinas-SENASA No. 04-288/A). The weight of the animals was monitored daily during the experiment (Supplementary Figure 2). All experiments with animals were performed according to the Animal Welfare Guidelines of NIH (INIBIOLP’s Animal Welfare Assurance No A5647-01) and were approved by the Universidad Nacional de La Plata Committee on Laboratory Animals (CICUAL; Protocol #T09-01-2013).

### Stereotaxic injection

Eight-months old animals were randomly divided into two experimental groups: PBS group (N_PBS_ = 9), which received an injection of 0.01 M pH 7.4 saline phosphate buffer solution; and RAd-IGF1 group (N_RAd-IGF1_ = 13), which received an injection of recombinant adenovirus carrying rat IGF1 therapeutic gene. Rats were anesthetized with ketamine (40 mg/kg) plus xylazine (8 mg/kg) and positioned on a stereotaxic instrument. Hypothalamic bilateral injections were performed by infusion of 4 μl per side of PBS or the suspension of RAd-IGF1, containing 10^10^ plaque-forming units (pfu) [42]. For injections, the tip of a 26G needle fitted to a 10 μl Hamilton syringe was positioned at the following coordinates with respect to the bregma: −3.0 mm anteroposterior, −9.1 ventral, ± 0.6 lateral [43].

### Determination of stage of estrous cycle

Vaginal secretion was collected daily (in the morning) by inserting the tip of a glass pipette into the rat’s vagina with a few drops of physiological solution (0.85% w/v NaCl), taking care not to do it deeply. A drop of vaginal fluid was smeared on a glass slide, and the unstained material was observed under a light microscope, with a 40× phase-contrast objective. The stage of the estrous cycle was determined by the cellular composition of the smears [44]. Vaginal smears were performed from 30 days before the beginning until the end of the treatment.

### Hormone measurement

Throughout the entire experimental period, weekly blood samples were obtained from tail vein of all rats. Serum was obtained and kept at −20°C until hormonal measurements. The concentrations of estradiol (E2) (17β-Estradiol high sensitivity ELISA kit; Catalog #: ADI-900–174) and luteinizing hormone (LH) (LH ELISA KIT; Catalog #: ENZ-KIT107) were measured using ELISA kits (Enzo Life Science) following the protocols provided by the manufacturer.

### Ovarian histology

Both ovaries of all females were fixed in 10% formalin solution and processed with histological conventional technique of paraffin embedded and hematoxylin and eosin staining [45]. Ten slices of each female ovarian was analyzed and their general histological characteristics were described.

### Immunohistochemistry

To perform the immunohistochemical staining of the brains, the rats were placed under deep anesthesia and transcardially perfused with paraformaldehyde 4% in PB 0.1M (pH 7.4) fixative. The brains were removed and stored in paraformaldehyde 4% in PB 0.1M (pH 7.4) overnight at 4°C. The brains were stored in cryoprotective solution (30% v/v ethylene glycol, 30% w/v sucrose, 0.1M PB, pH 7.4) at −20°C until used. Brains were sectioned coronally in 40 μm-thick sections with a microslicer (Vibratome, Leica) and one in every eight serial sections per rat was processed.

Sections were washed with PB 0.1M, treated with a solution of 3% v/v hydrogen peroxide in 50% v/v methanol to inhibit endogenous peroxidase, and then unspecific binding sites were blocked with blocking solution (PB 0.1M + 0.3% v/v Triton X-100 + 5% serum corresponding to the species in which the secondary antibody was made). Sections were incubated overnight at 4°C with the primary antibody. The following primary antibodies were used: polyclonal rabbit anti-GnRH (1:2000, Immunostar, Cat#: 20075), monoclonal mouse anti-Kiss-1 (1:200; Millipore; Cat #: MABC60), polyclonal rabbit anti-Iba1 (1:1000; Wako; Cat#: CTG2683), polyclonal rabbit anti-GFAP (1:1000; Dako; Cat#: Z0334). Sections were then washed and incubated for 2 hours at room temperature with the corresponding biotinylated secondary antibody (goat anti-rabbit 1:1000, Vector, Cat#: BA-1000; goat anti-mouse 1:1000, Thermo-Fisher, Cat#: 31800). Avidin-biotin-complex amplification system (ABC; 1:500; Thermo Fisher Scientific) was used for the detection of the secondary antibody, and the reaction product was revealed by incubating the sections with 3,3-diaminobenzidine (Sigma-Aldrich) and 0.01% hydrogen peroxide in PB 0.1M. Then, sections were mounted on gelatinized slides, dehydrated, covered with mounting medium (Canada balsam) and used for image analysis. To avoid inter-assay variations, experiments were run in parallel and non-specific staining was discarded by incubation of tissue without primary antibodies.

For the double staining of Iba1/MHCII, sections were incubated 48 h at 4°C with the following primary antibodies: polyclonal rabbit anti-Iba1 (1:1000; Wako; Cat#: CTG2683) and monoclonal mouse anti-MHCII (1:200; Serotec; Cat#: MCA46). After three washes, sections were incubated for 2 h with goat Alexa 488 conjugated anti-mouse (1:1000; Jackson ImmunoResearch, Cat#: 115-545-166) together with goat Alexa 568 conjugated anti-rabbit (1:1000; Abcam, Cat#: ab175471). Then, sections were mounted on gelatinized slides with mounting medium (Vectashield with DAPI, Vector) and used for image analysis.

For SA-β-Gal staining, sections were incubated overnight at 37°C in the dark with SA-β-Gal staining solution (Senescence β-Galactosidase Staining Kit, Cell Signaling Technology, Cat#: 9860). After incubation, sections were washed and DAB staining was continued as mentioned previously.

### Morphometric analysis

Number of Iba1 and kisspeptin immunopositive cells in the anteroventral periventricular nucleus (AVPV) and arcuate nucleus (ARC) and GnRH immunopositive cells in the preoptic area (PoA) was assessed. Areas of interest were defined in accordance with the rat brain atlas of Paxinos and Watson [43]. Iba1 immunoreactive cells were manually quantified on the computer display using Image-Pro Plus software (MediaCybernetics V5.1), and cell count is expressed as total number. GnRH and kisspeptin immunoreactive cells were manually quantified, according to the optical dissector method, using a counting frame of 123 × 123 μm at 400× magnification. A total of 10–20 counting frames per rat were analyzed (minimum of five sections). Cells intersecting the exclusion boundaries of the counting frame were not counted. Cells counts are expressed as number/mm^2^. For stereological analysis, we used an Olympus BX-51 microscope attached to an Olympus DP70 CCD video camera (Tokyo, Japan). Iba1 and MHCII immunopositive cells were manually quantified on pictures of individual focal planes using multi-point tool of Fiji-ImageJ software. Cells number was expressed as colocalization rate. For each animal, a minimum of two sections per area of interest, and at least 100 Iba1 immunopositive cells were quantified. The images were obtained with an Olympus FV1000 confocal microscope and the same exposure time settings were used for all the pictures.

### Statistical analysis

The data are represented as the mean ± SD. To evaluate the normality of the data we used the Kolmogórov-Smirnov and Shapiro-Wilk tests and to evaluate the homoscedasticity we used the Levene and Brown–Forsythe tests. Statistical differences in the frequency of cycles serum hormone levels were determined by 2-way ANOVA followed by Bonferroni’s post hoc test. Statistical differences in quantification of double positive cells (Iba1/MHCII) and number of GnRH, kisspeptin and Iba1 positive cells were determined by the Student’s *t* test when the SDs did not differ significantly, otherwise, the Welch’s approximate *t* estimator was used. Statistical analysis was performed by using the software GraphPad Prism 8 (GraphPad Software) and GPower 3.1 (Universität Kiel, Germany). *P*-values < 0.05 were considered to be significant.

## Supplementary Materials

Supplementary Figures
