# Supplementary material for: IGF1 gene therapy in middle-aged female rats delays reproductive senescence through its effects on hypothalamic GnRH and kisspeptin neurons
Source: Aging (Albany NY). 2022 Nov 1;14(21):8615–32. doi: 10.18632/aging.204360 (PMC9699762; doi:10.18632/aging.204360)
Supplement: Supplementary Figures [file aging-14-204360-s001.pdf]

SUPPLEMENTARY FIGURES

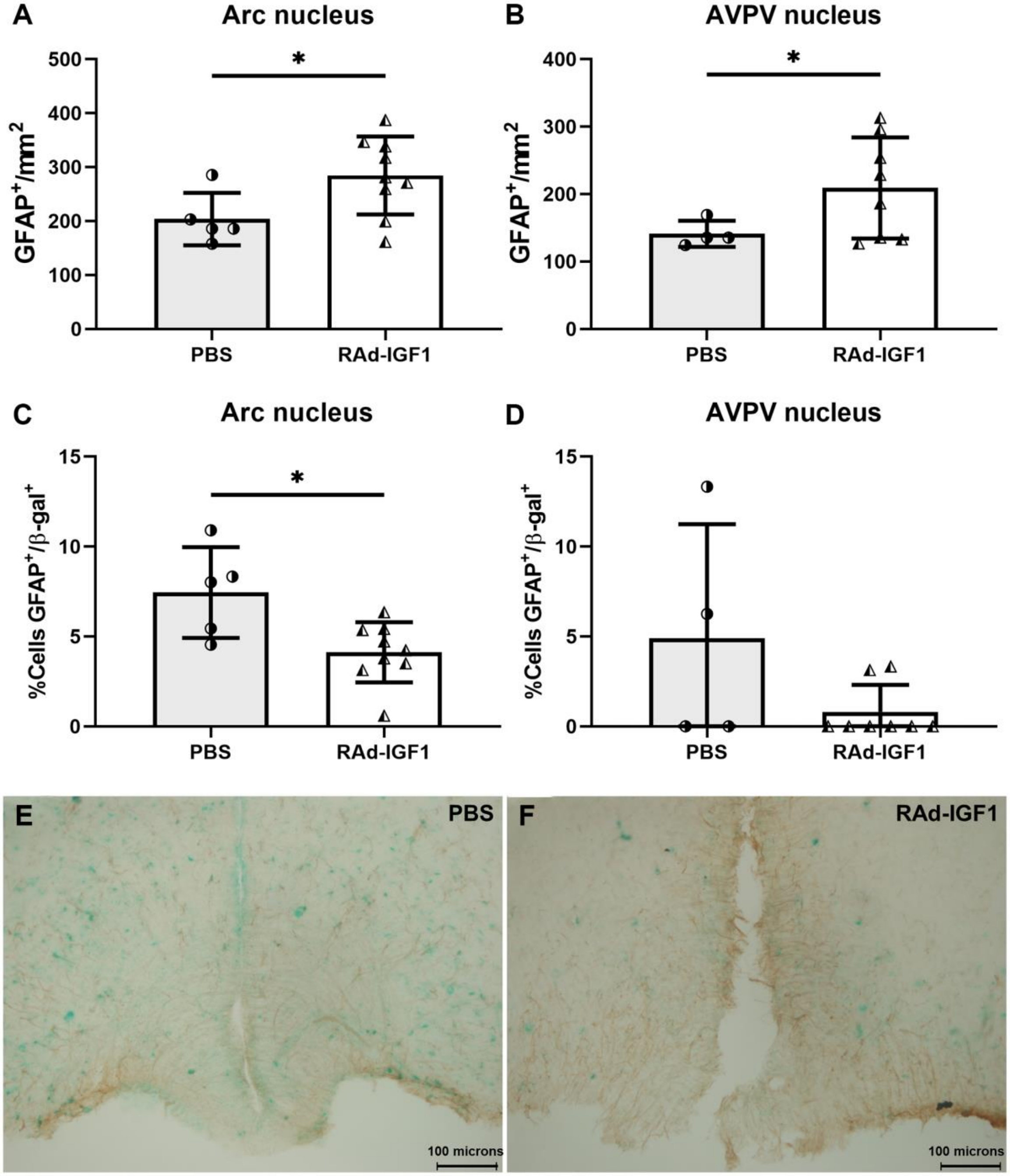

Supplementary Figure 1. IGF1 gene therapy effect on the GFAP immunopositive cells. (**A** and **B**) Quantification of GFAP immunopositive cells. (**C** and **D**) Percentage of astrocytes presenting dual-label for GFAP and SA-β-Gal (blue) staining. Error bars represent SD (Arcuate: $N_{RAd\text{-}IGF1} = 9$; $N_{PBS} = 5$. AVPV: $N_{RAd\text{-}IGF1} = 8$; $N_{PBS} = 4$). *t* test with Welch's correction was used. Asterisks indicate significant ($^{*}p < 0.05$) differences. (**E** and **F**) Representative example of GFAP/ SA-β-Gal double staining (Total magnification of 200×; scale bars: 100 microns).

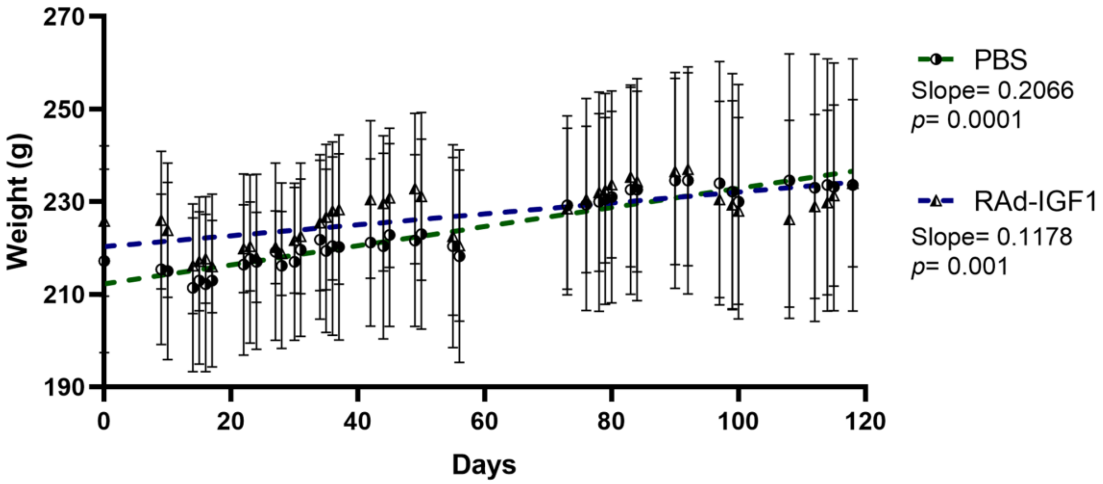

Supplementary Figure 2. Body weight curve of both groups of rats. Two-way ANOVA with repeated measures using the day and group as variable showed that during the 120 days experiment, differences in body weights between the control (PBS) and treatment (Rad-IGF1) groups were not significant. Error bars represent SD ($N_{RAd-IGF1}$ = 13; N_{PBS} = 9).
